# Creating a Public Data Resource: The HRS-O*NET Linkage

**DOI:** 10.1093/geroni/igab046.886

**Published:** 2021-12-17

**Authors:** Rebekah Carpenter, Dawn Carr, Brooke Helppie-McFall, Julia Beckel

**Affiliations:** 1 Florida State University, Tallahassee, Florida, United States; 2 University of Michigan, Ann Arbor, Michigan, United States; 3 Colorado State University, Fort Collins, Colorado, United States

## Abstract

Few longitudinal studies provide detailed information about the characteristics of the jobs older workers engage in, limiting the ability to evaluate the potential consequences of extended working lives. In this session, we introduce a new project linking the 2019 O*NET taxonomy and corresponding data to the Health and Retirement Study for public use. We describe the procedures taken to develop an O*NET linkage to be released to HRS users in the form of a publicly available data file, allowing aging researchers to evaluate detailed aspects of occupations in the 50+ population. We explain the types of variables that will be made available in the O*NET-HRS occupation project, and provide examples for how the measures can be used in longitudinal HRS studies.

